# Accumulation patterns of intracellular salts in a new halophilic amoeboflagellate, *Euplaesiobystra salpumilio* sp. nov., (Heterolobosea; Discoba) under hypersaline conditions

**DOI:** 10.3389/fmicb.2022.960621

**Published:** 2022-08-05

**Authors:** Hyeon Been Lee, Dong Hyuk Jeong, Jong Soo Park

**Affiliations:** Department of Oceanography, Kyungpook National University, Daegu, South Korea

**Keywords:** halophile, Heterolobosea, hypersaline, intracellular salt, osmoregulation, protozoa

## Abstract

Halophilic microbial eukaryotes are present in many eukaryotic lineages and major groups; however, our knowledge of their diversity is still limited. Furthermore, almost nothing is known about the intracellular accumulation of salts in most halophilic eukaryotes. Here, we isolate a novel halophilic microbial eukaryote from hypersaline water of 134 practical salinity units (PSU) in a solar saltern. This species is an amoeboflagellate (capable of the amoeba-flagellate-cyst transformation) in the heterolobosean group and belongs to the genus *Euplaesiobystra* based on morphological data and 18S rDNA sequences. However, the isolate is distinct from any of the described *Euplaesiobystra* species. Especially, it is the smallest *Euplaesiobystra* to date, has a distinct cytostome, and grows optimally at 75–100 PSU. Furthermore, the phylogenetic tree of the 18S rDNA sequences demonstrates that the isolate forms a strongly supported group, sister to *Euplaesiobystra hypersalinica*. Thus, we propose that the isolate, *Euplaesiobystra salpumilio*, is a novel species*. E. salpumilio* displays a significantly increased influx of the intracellular Na^+^ and K^+^ at 50, 100, and 150 PSU, compared to freshwater species. However, the intracellular retention of the Na^+^ and K^+^ at 150 PSU does not significantly differ from 100 PSU, suggesting that *E. salpumilio* can extrude the Na^+^ and K^+^ from cells under high-salinity conditions. Interestingly, actively growing *E. salpumilio* at 100 and 150 PSU may require more intracellular accumulation of Na^+^ than the no-growth but-viable state at 50 PSU. It seems that our isolate displays two salt metabolisms depending on the tested salinities. *E. salpumilio* shows a salt-in strategy for Na^+^ at lower salinity of 100 PSU, while it displays a salt-out strategy for Na^+^ at higher salinity of 150 PSU. Our results suggest that the novel halophilic *E. salpumilio* fundamentally uses a salt-out strategy at higher salinities, and the accumulation patterns of intracellular salts in this species are different from those in other halophilic microbial eukaryotes.

## Introduction

Hypersaline water (>40 practical salinity units; PSU) is considered an extreme environment to all three domains of life on Earth (Javor, 1989). The best-known hypersaline sites are lakes and lagoons that develop in warm and/or dry coastal regions due to high evaporation rates, but these extreme habitats are found in all continental areas on Earth as well as deep-sea basins in the oceans. These habitats accommodate halophilic/halotolerant organisms that have a variety of osmoregulation systems to manage homeostasis under extreme conditions (Javor, 1989; Harding and Simpson, 2018). Halophilic prokaryotes and eukaryotes that can grow optimally at 50 PSU or higher undergo salt adaptation processes, either ‘salt-in’ or ‘salt-out’, to thrive in hypersaline environments (Oren, 2008). Many halophilic prokaryotes have adopted the ‘salt-in’ strategy, which involves the intracellular accumulation of ions (e.g., K^+^) and the evolution of highly acidic proteins in order to reach an osmotic equilibrium with the environment (Lanyi, 1974). In contrast, the ‘salt-out’ strategy involves the intracellular accumulation of compatible solutes (e.g., glycerol, betaine, and hydroxyectoine), effectively displacing excessive salt ions from cells. This strategy is used by various halotolerant/halophilic prokaryotes, as well as halophilic fungi and the alga *Dunaliella* (Roberts, 2005; Plemenitaš et al., 2014; Gunde-Cimerman et al., 2018). It is inferred indirectly for other halophilic eukaryote taxa as well (e.g., *Halocafeteria seosinensis* and *Pharyngomonas kirbyi*, see Harding et al., 2016). It is unclear, however, whether these phylogenetically isolated studies reveal a general pattern, and it has been suggested, based on indirect inference, that some halophilic eukaryotes may instead employ a ‘salt-in’ strategy (Foissner et al., 2014). In short, there is a compelling need for direct information on salt management in halophilic taxa from across the tree of eukaryotes (Harding and Simpson, 2018; Weinisch et al., 2018). This is especially true of phagotrophic eukaryotes, which are free-living protozoans.

The taxon Heterolobosea *sensu lato* comprises morphologically and physiologically diverse protists found in freshwater, marine, and extreme habitats (Pánek et al., 2017; Harding and Simpson, 2018). Heterolobosea is regarded as a crucial group to better understand adaptation in halophilic/halotolerant eukaryotes because it includes several contrasting clades of these organisms (Kirby et al., 2015; Harding and Simpson, 2018; Jhin and Park, 2019). Recently, several studies have demonstrated that many halophilic/halotolerant eukaryotes (e.g., *Aurem*, *Euplaesiobystra*, *Percolomonas*, *Pharyngomonas*, *Pleurostomum*, *Selenaion*, and *Tulameoba*) are novel genera belonging to Heterolobosea (Park et al., 2007, 2009, 2012; Park and Simpson, 2011; Jhin and Park, 2019; Tikhonenkov et al., 2019; Aucher et al., 2020; Carduck et al., 2021). In addition to expanding the known diversity of halophilic eukaryotes, these studies suggest that a more complex food web than previously thought may function in hypersaline environments (Lee et al., 2022a).

At least four clades in Heterolobosea are largely or exclusively halophilic, with *Euplaesiobystra* representing one of these clades (Park et al., 2009; Harding and Simpson, 2018; Jhin and Park, 2019; Aucher et al., 2020). Like many heteroloboseids, *Euplaesiobystra hypersalinica* is an amoeboflagellate that can change from an amoeboid form to a flagellate one (or vice versa) during its life cycle (Pánek et al., 2017; Harding and Simpson, 2018), whereas the second species, *Euplaesiobystra dzianiensis,* has not been observed in a flagellate form (Aucher et al., 2020). Park et al. (2009) formally described *Euplaesiobystra hypersalinica* based on an isolate from 293 PSU saline water in a Korean solar saltern, with the same species having been isolated by different researchers from ~140 PSU saline water in Portugal (T.A. Nerad, pers. comm.; see Park et al., 2009). A related 18S rDNA sequence has also been obtained from Hutt Lagoon, Australia (Park and Simpson, 2015). The more recently described *Euplaesiobystra dzianiensis* is a halotolerant species isolated from 52 PSU saline water in Dziani Dzaha, a small crater lake on a Caribbean Island (Aucher et al., 2020). Thus, the genus *Euplaesiobystra* is present in hypersaline waters worldwide. Recently, Illumina sequencing targeting the V9 region of 18S rDNA revealed six unclassified *Euplaesiobystra* sequences in the Eui-Seong solar saltern, Republic of Korea (Lee et al., 2022a). This result suggests that *Euplaesiobystra* is more diverse than previously realized, and implies that several novel species of *Euplaesiobystra* are present at the solar saltern. Thus, these data motivated an investigation of the heterolobosean diversity in the hypersaline waters of the Eui-Seong solar saltern.

In the present study, we isolated an unclassified amoeboflagellate from high-salinity water (134 PSU) in the Eui-Seong solar saltern, Republic of Korea, and then investigated the morphology and phylogenetic trees of 18S rDNA sequences to determine a suitable taxonomic assignment. Furthermore, we determined the salinity (3 to 300 PSU) and temperature (5 to 55°C) regimes required for the growth of this isolate. Finally, we assessed the accumulation patterns of intracellular sodium (Na^+^) and potassium (K^+^) in culture media with 50 PSU, 100 PSU, and 150 PSU using CoroNa Green and ION Potassium Green-2 AM, which are specific fluorescence markers to detect directly the distribution of intracellular Na^+^ and K^+^, respectively (Park et al., 2009; Weinisch et al., 2018). We investigated the salt metabolism of the new species encountered, and hypothesized that our isolate might use a salt-out strategy of osmoadaptation based on our knowledge of halophilic/halotolerant eukaryotes. Moreover, because our isolate was found at 134 PSU, we hypothesized that the new species encountered (*Euplaesiobystra salpumilio* sp. nov.) was likely to be a true halophile, rather than a halotolerant.

## Materials and methods

### Isolation and cultivation

An amoeba strain ES010 was isolated from 134 PSU water collected in October 2020 from the Eui-Seong solar saltern, Taean, Republic of Korea (36°36′ 08.3″ N, 126°17′49.3″ E). A monoeukaryotic culture was established using manual single-cell picking with a micropipette. In brief, isolated single cells were inoculated into separate wells of a 24-well culture plate (30,024, SPL Life Sciences Co., Ltd., Pocheon, Republic of Korea) filled with 100 PSU salinity liquid media, which was prepared by the dilution of Medium V (300 PSU; 272 g NaCl, 7.6 g KCl, 17.8 g MgCl_2_, 1.8 g MgSO_4_ ·7H_2_O, and 1.3 g CaCl_2_ per liter water; see Park, 2012) with sterile double distilled water (DDW) plus Luria-Bertani broth (LB; final concentration of 0.5%), and incubated at 25°C for 7 days. LB supported the growth of prokaryotes, which were in turn prey for the isolated eukaryotic cells. Subsequently, cells of the ES010 isolate were transferred to and maintained in 50 ml tissue flasks (70,125, SPL Life Sciences Co., Ltd., Pocheon, Republic of Korea) containing 10 ml of Medium V (100 PSU) with 0.5% LB (final concentration) and autoclaved barley grains. The strain ES010 was incubated at 25°C and sub-cultured every 3 weeks.

### Molecular sequencing and phylogeny

Nucleic acids from isolate ES010 were extracted using a DNeasy Blood and Tissue kit (Qiagen, Hilden, Germany), according to the manufacturer’s instructions. The primers EukA (5′-AACCTGGTTGATCCTGCCAGT-3′) and EukB (5′-TGATCCTTCTGCAGGTTCACCTAC-3′) were used to amplify 18S rRNA gene sequences (Medlin et al., 1988). The reaction mixture (20 μl) contained 0.8 μl each of 10 μM stocks of primers EukA and EukB, 0.4 μl of a 2.5 mM dNTP-mix, 2 μl 10× PCR buffer (Mg^2+^ plus), 0.2 μl TaKaRa Taq™ polymerase (TaKaRa Bio Inc., Shiga, Japan), 13.8 μl of double distilled water, and 2 μl of extracted DNA template. DNA amplification was performed on a Biometra TRIO thermal cycler (Analytik Jena, Jena, Germany) with the PCR steps as follows: an initial denaturing step at 94°C for 5 min, followed by 40 cycles of 45 s at 94°C, 1 min of annealing at 55°C, and extension at 72°C for 3 min, with a final extension step for 20 min at 72°C. Amplicons were cloned into a pGEM-T Easy vector. Five positive clones were partially sequenced using the vector sequencing primer T7, and one positive clone was completely sequenced using the vector sequencing primer SP6 and various eukaryotic internal sequencing primers (e.g., Euk345F, Zhu et al., 2005, and Euk1139R, El-Sherry et al., 2013). The 18S rRNA gene sequence from the isolate ‘ES010’ was deposited in GenBank under the accession number ON561780.

The 18S rRNA gene sequences from 68 representative heterolobosean species, including isolate ES010, plus sequences from 16 representative non-heterolobosean species selected as outgroups, were used for phylogenetic analysis (the seed alignment originated from Tikhonenkov et al., 2019; Carduck et al., 2021). The dataset was aligned using the MAFFT program v.7 (Katoh and Standley, 2013) and masked by eye, with 1,348 sites retained. Maximum likelihood trees were estimated using the IQ-TREE v.1.6.12. The GTR + F + I + G4 model of sequence evolution was selected using the best-fit model test option (−m TEST, Minh et al., 2013; Flouri et al., 2015; Nguyen et al., 2015). Statistical support was assessed by bootstrapping with 1,000 replicates. The Bayesian analysis was conducted using the MrBAYES 3.2.7a (Ronquist et al., 2012) with two independent runs, each with four chains running for 5,000,000 generations with the default heating parameter (0.1) and sampling frequency (0.01). A conservative 30% burn-in was used; the average standard deviation of split frequencies for the last 70% of generations was <0.01.

A phylogenetic analysis was also performed with twelve *Euplaesiobystra*-like 18S rRNA gene sequences, including the isolate ‘ES010’ and six environmental sequences from a Korean solar saltern (Lee et al., 2022a), and two *Heteramoeba* sequences as outgroups. A total of 1,348 sites were included in this analysis. A phylogenetic tree was constructed as described above, except that that the Bayesian analysis was run for 20,000,000 generations.

### Microscopy

Live amoebae, flagellates, and cysts mounted on glass slides were observed with differential interference microscopy using a Leica DM5500B microscope equipped with a DFC550 digital camera (Leica, Wetzlar, Germany). Images were taken with this digital camera, and the dimensions of the organisms were measured using the LAS v.4.12 software (Leica Microsystems Ltd. Heerbrugg, Switzerland).

For scanning electron microscopy, cultures (800 μl) were fixed by adding 200 μl of 25% v/v glutaraldehyde (electron microscopy grade, Sigma-Aldrich, St. Louis, MO, United States), and incubated overnight at 4°C. Fixed cells were centrifuged at 5,000 × *g* for 30 min, and then 800 μl of the supernatants were discarded. Concentrated cells (200 μl) settled down on the glass coverslips coated with 1% poly-L-lysine for 1 h. Cells were then dehydrated using a graded ethanol series (50–100%). The glass coverslips were dried by adding a few drops of heated 2% ILs (1-Butyl-3-methylimidazolium tetrafluoroborate, Sigma-Aldrich, St. Louis, MO, United States) at 40°C onto the coverslip surface, followed by spinning at 3,000 × *g* for 5 s. The fixed cells were coated with platinum using an ion-sputter system. The specimens were subsequently examined under an SU8220 field emission scanning electron microscope (Hitachi, Tokyo, Japan).

### Salinity and temperature ranges for growth

To estimate the salinity ranges for the growth of the isolate ‘ES010’, we performed an experiment using media of 10 different salinities ranging from 3 to 300 PSU, as reported by Park and Simpson (2010, 2011). All treatments were performed in triplicates. Medium V (990 μl) with a range of salinities (3–300 PSU) was inoculated with 10 μl of actively growing stock culture (100 PSU media with an autoclaved barley grain) and incubated in the dark at 35°C for 7 days. Culture viability was assessed daily by observing actively moving cells in 10 μl of the culture using phase-contrast microscopy. The salinity range tolerated for growth was confirmed by a serial transfer into fresh media with the same salinity (990 μl of media and inoculum of 10 μl), and re-examining the culture daily for active cells for 7 days.

To determine the optimal temperature for the growth of the isolate ‘ES010’, culture tubes including 990 μl of 100 PSU Medium V were inoculated with 10 μl of actively growing stock culture (total 1 ml), with an autoclaved barley grain and then incubated in the dark at 5, 15, 25, 35, 37, 40, 45, 50, and 55°C. Actively moving cells were checked daily in 10 μl of culture for 7 days. Growth was confirmed by transferring a sample of the isolate into fresh media, followed by incubation at the same temperature and microscopic observation as described above.

### Intracellular ion accumulation assay

To assess whether the isolate ‘ES010’ accumulates sodium (Na^+^) or potassium (K^+^) ions inside cells, an intracellular ion accumulation assay was conducted with Na^+^- and K^+^-specific fluorescence dyes (Park et al., 2009; Weinisch et al., 2018). The experiment was performed on cultures of ‘ES010’ incubated at 50, 100, and 150 PSU for 7 days. In addition, the freshwater species *Naegleria neojejuensis* was used as a negative control (Khwon and Park, 2018). The cells were concentrated by centrifugation at 5,000 × *g* for 30 min. To identify intracellular Na^+^ and K^+^, viable cells after concentration were stained with of 50 μM CoroNa Green (final concentration, DMSO mixed with CoroNa Green, AM, Invitrogen™, Thermo Fisher Scientific, Waltham, MA, United States) or 50 μM ION Potassium Green-2 (final concentration, DMSO mixed with ION Potassium Green-2 AM, ab142806, Abcam, Cambridge, United Kingdom), respectively, and incubated in the dark at room temperature (~20°C) for 3 h. Cells were washed twice with sterile culture medium (same salinity as that used for growth) to remove excess fluorescent dye. The stained cells were mounted on glass slides and observed under an Olympus BX53 fluorescence microscope equipped with a specific filter set (Ex/Em = 492/516 nm for CoroNa Green; Ex/Em = 526/546 nm for ION Potassium Green). Images were taken with a digital camera at 5 s intervals for 10 s, and the exposure time for each frame was 500 ms using the Olympus cellSens Standard v.2.3 software (Olympus Corporation, Tokyo, Japan).

The relative fluorescence intensity of ten cells at each salinity level was measured using the open-source software FIJI (Schindelin et al., 2012). The area of the cytoplasm (A_c_), integrated density of the cytoplasm (I_c_) and mean fluorescence of four different background readings (F_b_) were determined to obtain the corrected total cell fluorescence of the cytoplasm (CTCF_c_). The CTCF_c_ was calculated as follows:


$${\mathrm{CTCF}}_{c}=I_{c}-\left(A_{c}\times F_{b}\right)$$

CTCF_c_ was normalized (CTCF_norm_) to the measured area of the cytoplasm as follows:


$${\mathrm{CTCF}}_{\mathrm{norm}}={\mathrm{CTCF}}_{c}/A_{c}$$

We excluded vacuole-like structures inside cells from the CTCF_norm_ as described by Weinisch et al. (2018).

A Kolmogorov–Smirnov test was carried out to assess the normality of the dataset, and Levene’s test was used to check for homogeneity of variance. To reveal statistically significant differences between the different salinities, one-way ANOVA was performed with a Games–Howell test for a nonparametric *post hoc* analysis and a Turkey’s honestly significant difference (HSD) test for a parametric *post hoc* analysis. All statistical analyses were performed using SPSS v25.0.

## Results

### Light microscopy and scanning electron microscopy

**Figure 1:**
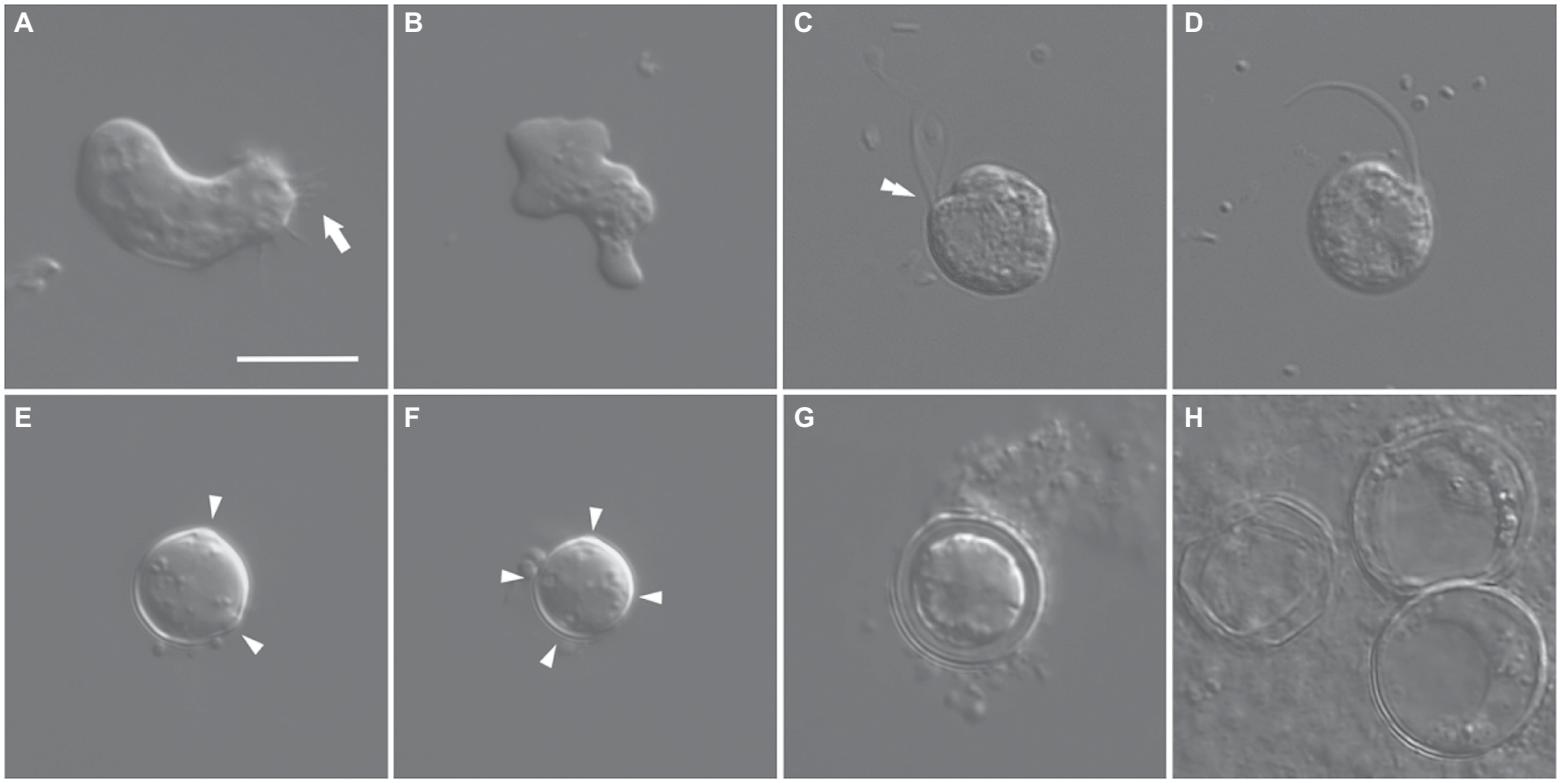
Differential interference contrast micrographs of *Euplaesiobystra salpumilio* sp. nov. **(A,B)** Amoebae (trophozoites), showing size and shape (monopodial or polypodial), and pseudopod formation. Arrow in **(A)** denotes uroidal filaments. **(C,D)** Flagellate phase, with double-arrowhead denoting rostrum. **(E–H)** Cysts with a round shaped, four cyst pores, and double wall. **(G)** Empty cyst **(H)**. Arrowheads in **(E,F)** denote cyst pores. Scale bar: 10 μm for all figures.

The trophozoites of the isolate ES010 were limax and monopodial or polypodial amoebae with eruptive pseudopodia (Figures 1A,B). The average length and width of the amoebae were 17.6 μm (range: 11.5–23.2 μm) and 9.2 μm (range: 5.1–14.5 μm), respectively (*n* = 30). The average length-to-width ratio was approximately 2.1 (range: 1.2–3.2). Some trophozoites had fine and short uroidal filaments at the posterior end of the cells (arrow in Figure 1A). The cells had a single nucleus with a central nucleolus and contained several food vacuoles and rounded inclusions (Figures 1A,B).

A flagellate form was occasionally observed in the liquid culture of the ‘ES010’ strain (Figures 1C,D). The flagellates showed a nearly spherical shape (9–10 μm in diameter) with two equal-length flagella. The flagellar length was about 1.5–2.0 times the body size. The flagellates had a shallow groove (cytostome) and a small rostrum in the anterior part of the cell (double-arrowhead in Figure 1C). When some flagellates swam, the anterior flagellum was directed anteriorly, while the posterior flagellum trailed posteriorly. Furthermore, when some flagellates rotated counter-clockwise, the anterior flagellum beat flexibly, while the posterior flagellum was wrapped in the cell body.

**Figure 2:**
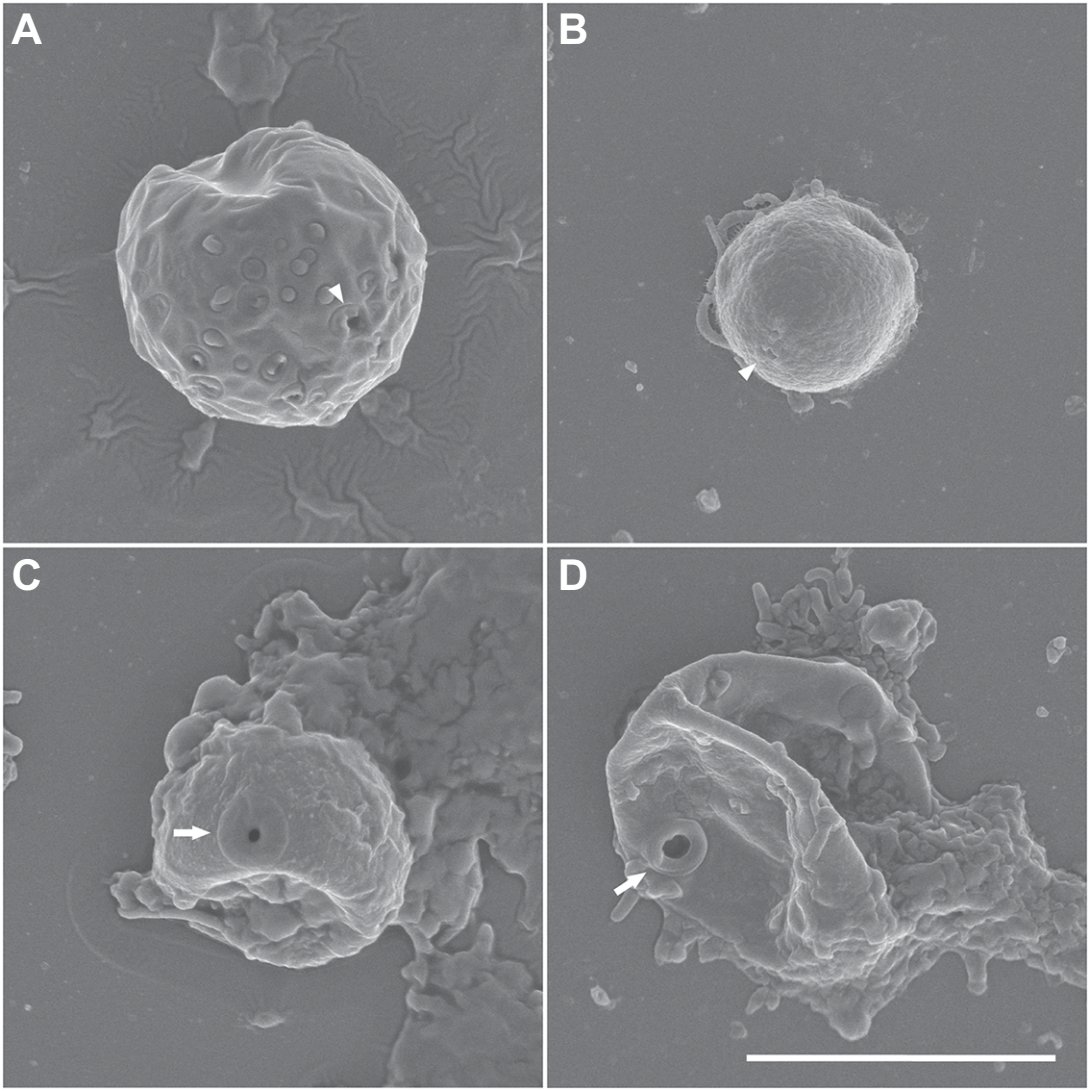
**(A–D)** Scanning electron micrographs of *Euplaesiobystra salpumilio* sp. nov. cysts, showing cyst shape and pore. Arrow represents cyst pore. **(C,D)** Empty cysts following excystation. Scale bar: 5 μm for all figures.

Cysts were usually spherical-shaped (Figures 1E–H), with the average diameter of 12.5 μm (range: 8.3–19.3 μm, *n* = 30), and the thickness of the cyst walls ranged from 0.6 to 2.3 μm, consisting of thick endocyst and thin ectocyst walls (Figure 1G). The ectocysts usually had a smooth outline. The cysts had 2–4 visible pores that did not penetrate the whole cyst wall. Some cysts had no cytoplasm, presumably due to excystment (Figure 1H). Scanning electron micrographs showed that the surface of the cysts had a granular appearance, and some images showed small cyst pores (arrowheads in Figures 2A,B), which were not observed by light microscopy. The excysted cysts displayed a conspicuous rounded pore surrounded by thickened ectocyst material (Figures 2C,D).

### Molecular phylogeny

**Figure 3:**
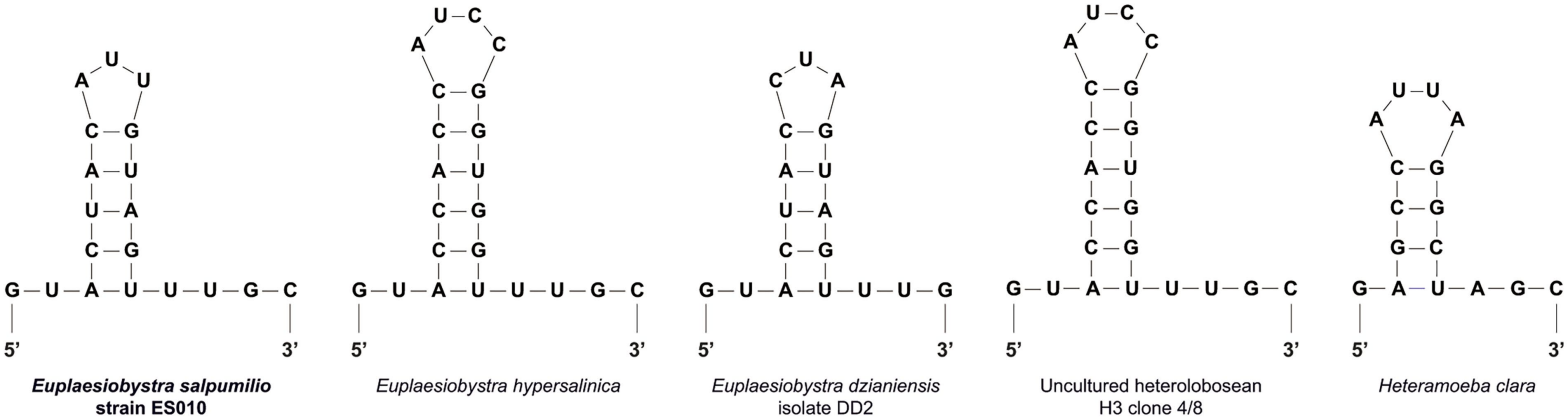
Putative secondary structures of the helix 17_1 in the 18S rRNA molecules of the *Euplaesiobystra* group and *Heteramoeba clara*. Note that all heterolobosean species except *Pharyngomonas* strains has the helix 17_1 feature.

The 18S rRNA gene sequence of isolate ES010 was 2,659 bp long, and included a group I intron of 791 bp in length after position 552 in the amplified sequence. The most similar sequences returned by a BLASTN search of GenBank were heteroloboseids, including two from the amoeboflagellate *Euplaesiobystra hypersalinica* (= *Plaesiobystra hypersalinica;* AF011459 and FJ222604) and one from the amoeba *Euplaesiobystra dzianiensis* (MN969059), which has no known flagellate form at present (Aucher et al., 2020). These *Euplaesiobystra* sequences were 80.6–81.2% identical to ES010. The ES010 sequence included a helix 17_1 structure (Figure 3), which was a unique insert within the 18S rDNA sequences of all heterolobosean taxa, except for Pharyngomonadea (Nikolaev et al., 2004; Park and Simpson, 2011; Harding et al., 2013).

**Figure 4:**
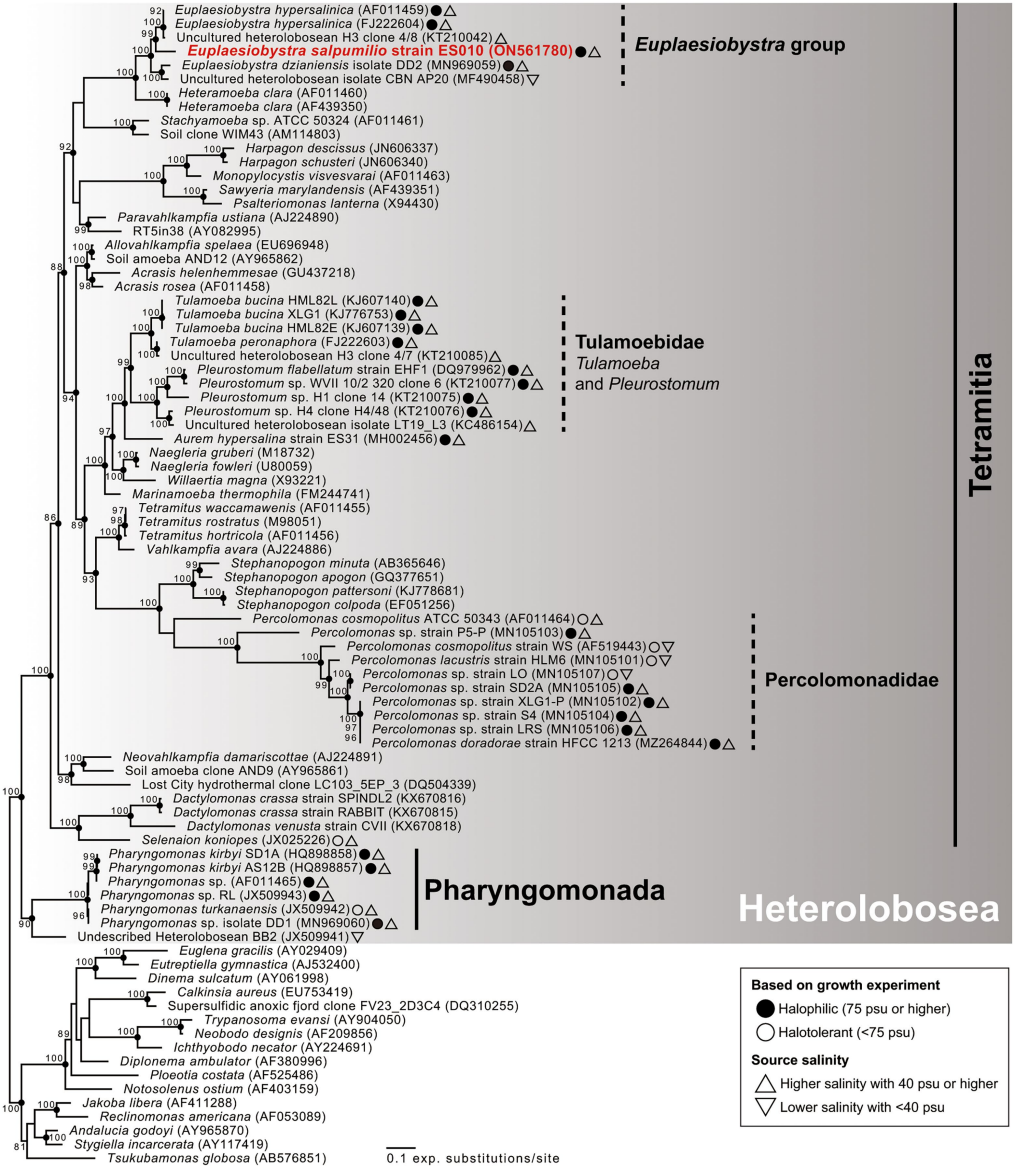
Maximum likelihood phylogenetic tree of 18S rRNA gene sequences showing the phylogenetic position of *Euplaesiobystra salpumilio* sp. nov. relative to 68 heterolobosean taxa and 16 species outgroups (i.e., Euglenozoa, Jakobida, and *Tsukubamonas globosa*). Bootstrap support values (>80%) are shown at the nodes. Solid circles represent a Bayesian posterior probability of 1 (posterior probability <0.95 not shown).

**Figure 5:**
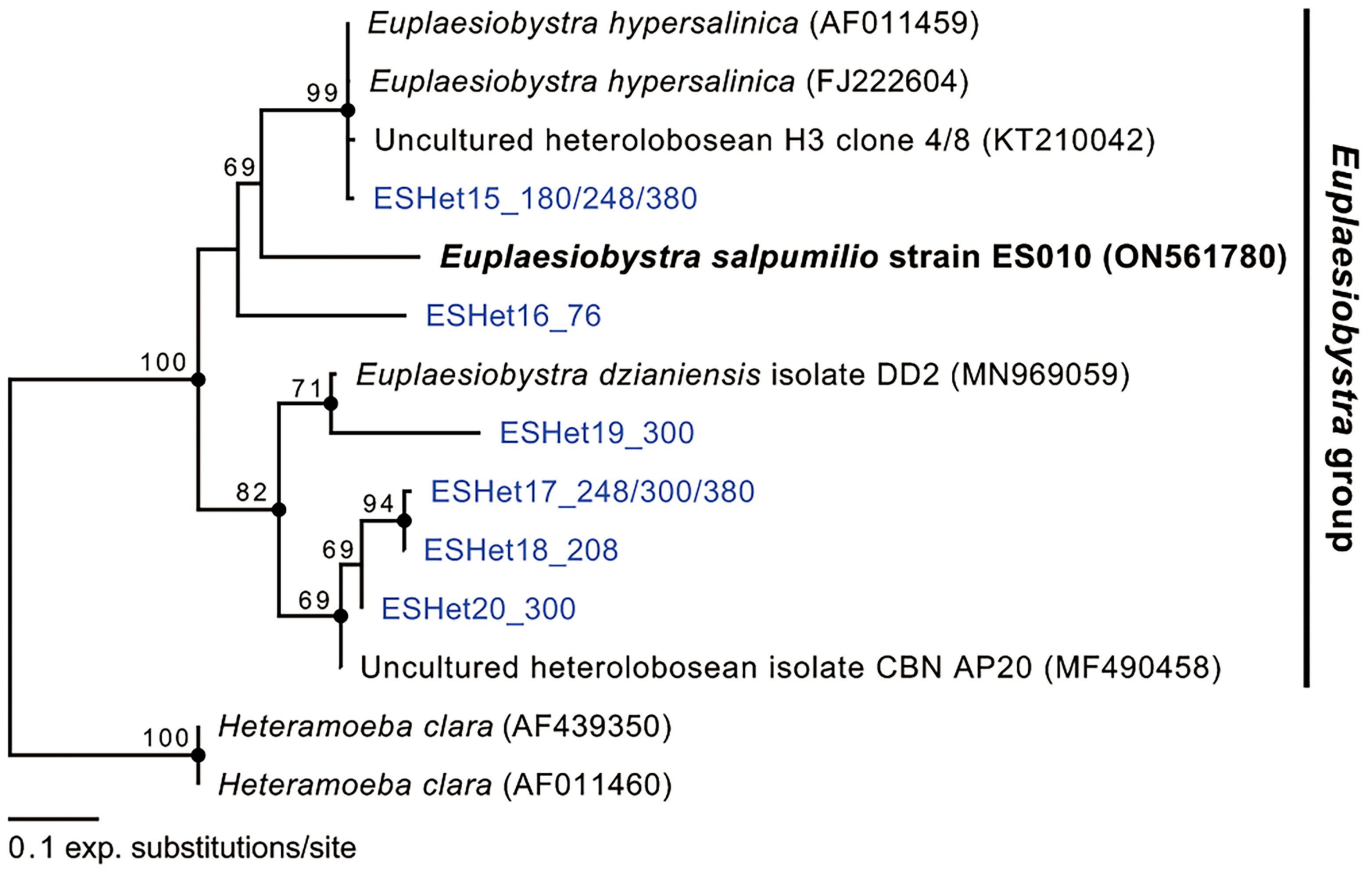
Maximum likelihood phylogenetic tree of *Euplaesiobystra* ingroup, including *Euplaesiobystra salpumilio* sp. nov. and six uncultured *Euplaesiobystra*-like sequences (blue) and *Heteramoeba clara*. Bootstrap support values (>65%) are shown at the nodes. Solid circles represent a Bayesian posterior probability of 1 (posterior probability <0.95 not shown).

Phylogenetic analyses of 18S rDNA sequences revealed that isolate ES010 was a new member of Tetramitia in Heterolobosea with maximum bootstrap support (ML [maximum likelihood]: 100%) and posterior probability (PP) of 1 (Figure 4). Furthermore, the genus *Euplaesiobystra* including isolate ES010 formed a monophyletic group with high bootstrap support (ML: 100%) and PP of 1. Isolate ES010 was more closely related to *E. hypersalinica* than to *E. dzianiensis* or any environmental sequences, with high bootstrap support (ML: 99%) and PP of 1 (Figure 4). However, ES010 was clearly a distinct lineage from the two *E. hypersalinica* sequences, with the uncultured sequence H3 forming a tight and robust clade to the exclusion of ES010 (ML: 100%; PP: 1). This basic position remained when six uncultured *Euplaesiobystra* sequences from a Korean solar saltern (hypersaline waters of 76 PSU to 380 PSU, Lee et al., 2022a) were included in the phylogenetic analyses, although with lower support for the sister group relationship between *E. hypersalinica* and ES010 (Figure 5). Thus, we propose isolate ES010 as a new species, *Euplaesiobystra salpumilio* sp. nov., based on morphology and molecular phylogeny (see below).

### Growth properties

The isolate ES010 could grow in a narrow range of salinity (75–150 PSU) at 35°C. The optimal salinity for growth in liquid culture was 75–100 PSU when barley grain was added to grow the indigenous prokaryotes (Table 1). This profile is similar to that of other halophiles rather than halotolerant organisms (Oren, 2008; Jhin and Park, 2019; see Discussion). The ES010 cultures did not show positive net growth at 50 or 200 PSU, but the cells remained viable state at these salinities for at least 7 days (data not shown). The isolate grew well at 25, 35, 37, and 40°C in a liquid culture of 100 PSU, and the highest density was observed at 35°C. The cultures died at 15°C (or lower) or at 45°C (or higher) after an incubation period of 7 days.

**Table 1:** Salinity ranges and maximum temperature tolerated for the growth of halophilic/halotolerant heteroloboseans.

		Salinity (‰)										Maximum temperature tolerated	References
Category	Species	3	15	30	50	75	100	150	200	250	300		
Obligate halophilic heteroloboseans	*Pleurostomum flabellatum*								+	+	++	45°C	Park et al. (2007)
	*Tulamoeba peronaphora*					+	+	++	++	+		43°C	Park et al. (2009)
	*Tulamoeba bucina* XLG1				+	+	++	++	+			n.d.	Kirby et al. (2015)
	*Pharyngomonas kirbyi* AS12B					+	++	++	+			40°C	Park and Simpson (2011)
	*Pharyngomonas kirbyi* SD1A						+	++	++	+		40°C	Park and Simpson (2011)
	*Aurem hypersalina*						+	++	+			At least 45°C	Jhin and Park (2019)
	*Euplaesiobystra hypersalinica*						+	++	++	+	+	50°C	Park et al. (2009)
	*Euplaesiobystra salpumilio* ES010					++	++	+				40°C	This study
Halotolerant heteroloboseans	*Euplaesiobystra dzianiensis* DD2		++	++	++	+						37°C	Aucher et al. (2020)
	*Selenaion koniopes*		+	++	++	+	+	+				40°C	Park et al. (2012)
	*Pharyngomonas turkanaensis*		+	++	++	+	+					n.d.	Park and Simpson (2016)

+, growth of relatively low density; ++, growth of relatively high density.

### Accumulation patterns of intracellular Na^+^ and K^+^

**Figure 6:**
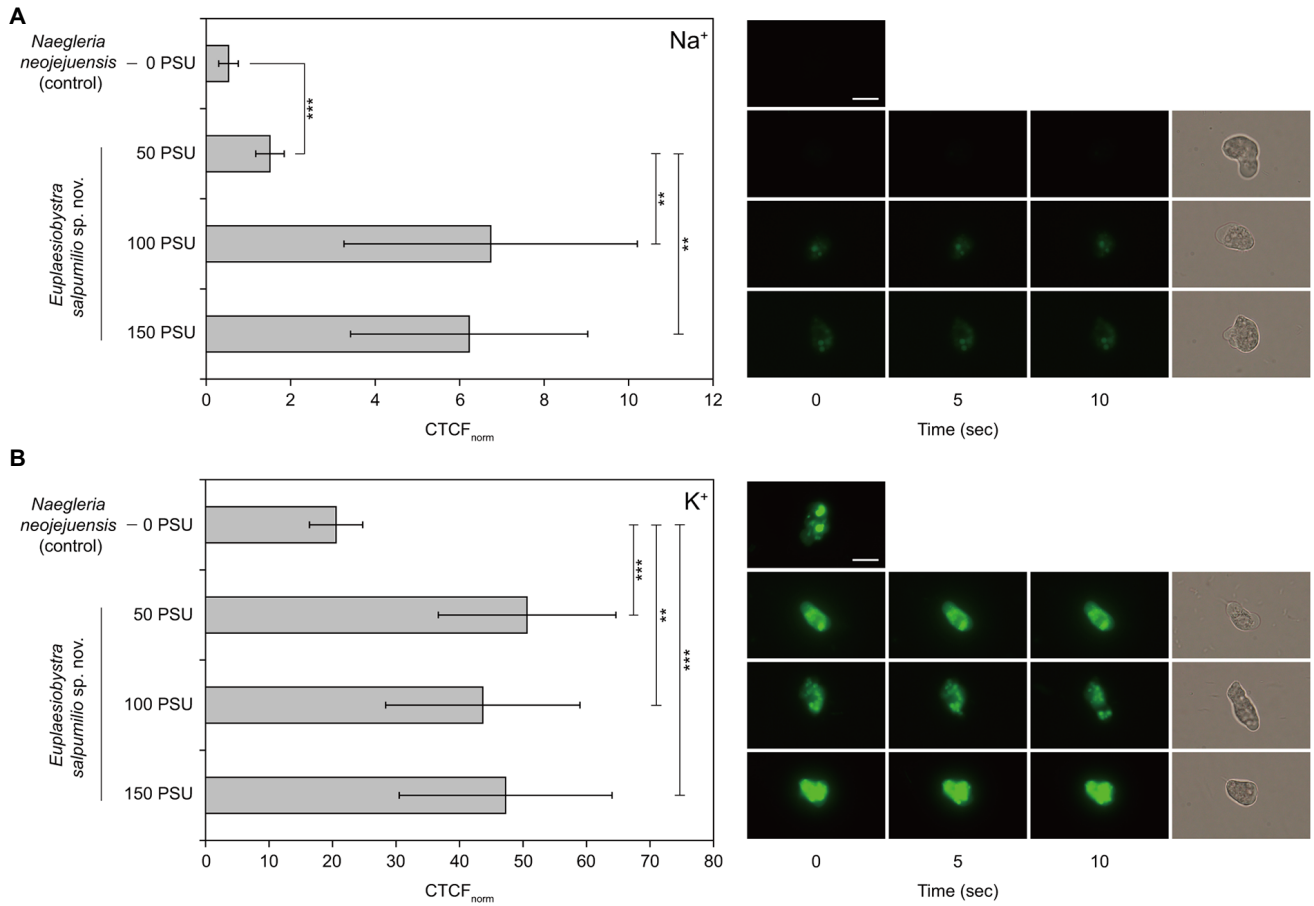
Normalized corrected total cell fluorescence of *Euplaesiobystra salpumilio* sp. nov. with **(A)** CoroNa Green (for Na^+^) and **(B)** ION Potassium Green (for K^+^) at increasing external salinities. The freshwater amoeba *Naegleria neojejuensis* was used as a negative control (see Khwon and Park, 2018). Note that fluorescence images were taken for 10 s at 5 s intervals, after which a light microscopy image was immediately obtained. Results shown represent the mean ± SD (*n* = 10). Data of CoroNa Green and ION Potassium Green were compared using ANOVA with Games–Howell and Turkey’s honestly significant difference analyses, respectively (**p < 0.01; ***p < 0.001). Scale bar: 10 μm for all microscopy images.

Only the fluorescence intensity of the cytoplasm, excluding vacuole-like structures, was considered to identify the accumulation patterns of intracellular Na^+^ and K^+^ (Weinisch et al., 2018). The CTCF_norm_ values associated with intracellular Na^+^ at external salinities of 50, 100, and 150 PSU were significantly higher than those of the freshwater amoeba ‘*Naegleria neojejuensis*’ growing in the absence of salts (Figure 6A). Furthermore, the CTCF_norm_ values of ES010 corresponding to intracellular Na^+^ at 100 PSU and 150 PSU were significantly higher than those at 50 PSU (*p* < 0.01). However, no significant difference was observed in CTCF_norm_ between the 100 PSU and 150 PSU groups (*p* > 0.05; Figure 6A). Similar to the patterns of intracellular Na^+^, CTCF_norm_ showed a significant increase in the intracellular K^+^ level of isolate ES010 at 50, 100, and 150 PSU compared to that of the freshwater amoeba *Naegleria neojejuensis* (Figure 6B). However, no significant difference was observed in CTCF_norm_ between external salinities (*p* > 0.05; Figure 6B). The brightest fluorescent areas of intracellular Na^+^ and K^+^ were observed in vacuole-like structures (Figure 6).

## Discussion

### Halophily of *Euplaesiobystra salpumilio*

Compared to the long history of literature on the halophily of prokaryotes, the study on the halophily of protozoa is much more limited, dating back to the last 15 years. This difference may be due to difficulties in successfully cultivating protozoa (Post et al., 1983). The most widely used definition of halophiles, which was reported by Kushner (1978), is based on yeast, fungi, and bacteria. He defines halophilic and halotolerant microorganisms as follows: extreme halophiles (growing best at 150–300 PSU), borderline extreme halophiles (growing best at 90–230 PSU), moderate halophiles (growing best at 30–150 PSU), slight halophiles (growing best at 10–30 PSU), and halotolerant microorganisms that do not absolutely require salts for growth. More recently, Oren (2008) defines halophiles as organisms that can grow best at 50 PSU or higher, and tolerate at 100 PSU or higher. Thus, *Euplaesiobystra salpumilio*, which can grow best at 75–100 PSU, is considered to be a true halophile by either definition.

### Taxonomic assignment

The morphology and life history of halophilic *E. salpumilio* sp. nov., are typical for heterolobosean amoeboflagellates, which are capable of transforming from amoebae into both flagellates and cysts. Based on contemporary studies with DNA sequence and morphological data, heterolobosean amoeboflagellates include some *Euplaesiobystra*, *Heteramoeba*, *Monopylocystis*, some *Naegleria*, *Oramoeba*, some *Pharyngomonas*, *Psalteriomonas*, *Pseudoharpagon*, *Stachyamoeba* (ATCC 50324), most *Tetramitus*, *Tulamoeba*, and *Willaertia* (Murase et al., 2010; De Jonckheere et al., 2011; Pánek et al., 2012, 2017; Harding and Simpson, 2018). *Tetramastigamoeba* (probably, *Willaertia*) and *Trimastigamoeba* are regarded as amoeboflagellates, but there are no DNA sequence data (Park et al., 2009; Pánek et al., 2012). Our organism, *E. salpumilio,* differs from *Monopylocystis*, *Pharyngomonas*, *Psalteriomonas*, *Pseudoharpagon*, most *Tetramitus*, and *Willaertia*, all of which transform into flagellates with four flagella (Robinson et al., 1989; Park et al., 2009; Park and Simpson, 2011; Pánek et al., 2012, 2014; Park, 2017). *Psalteriomonas lanterna* has four kinetids of four flagella each. Meanwhile, the flagellate form of *Oramoeba* can have two to ten flagella (De Jonckheere et al., 2011). *Monopylocystis*, *Psalteriomonas*, and *Pseudoharpagon* appear to be anaerobes, whereas our isolate is not, and *Tetramitus* and *Stachyamoeba* lack cyst pores (Murase et al., 2010; Pánek et al., 2017). The length of the cytostome in the flagellate phase of *Pharyngomonas* (>9 μm) is longer than that in *E. salpumilio*, whereas *Willaertia* has no cytostome (Patterson et al., 2000; Park and Simpson, 2011). *Naegleria* flagellates have two flagella, but lack a cytostome (Khwon and Park, 2018). *Tulamoeba* is isolated from hypersaline environments and has two flagella, but the flagellates show an elongated shape (Kirby et al., 2015) rather than the spherical shape of *E. salpumilio*. The cyst form also has a single pore with a characteristic large plug (Park et al., 2009), or no pores (Kirby et al., 2015).

Our isolate is more similar to *Euplaesiobystra* and *Heteramoeba* than to other amoeboflagellates. *Euplaesiobystra* and *Heteramoeba* possess round-shaped flagellates with two flagella during their life stages. However, these two taxa are distinguishable from each other based on their nuclear shape and cyst pores. *Euplaesiobystra* has a nucleus with a central nucleolus and has cyst pores, whereas *Heteramoeba* shows nucleolus scattered into large irregular patches and lacks cyst pores (Droop, 1962; Park et al., 2009). In these respects, our isolate resembles *Euplaesiobystra.* Moreover, all known *Euplaesiobystra* are isolated from hypersaline environments with >50 PSU, while *Heteramoeba* is isolated from ordinary marine environments (Droop, 1962; Park et al., 2009; Kirby et al., 2015; Aucher et al., 2020). Thus, we consider the isolate described in this study to be a member of *Euplaesiobystra* as was strongly confirmed by our molecular phylogenetic analysis (see below).

Prior to this study, the genus *Euplaesiobystra* has been identified three times in culture-dependent studies (Park et al., 2009; Aucher et al., 2020). *E. hypersalinica* is isolated from high-salinity waters with 140 PSU and 293 PSU in Portugal and Korea, respectively (Park et al., 2009), while *E. dzianiensis* is isolated from a water sample with 52 PSU obtained from the small lake Dziani Dzaha on Petite Terre island in the Caribbean (Aucher et al., 2020). *E. salpumilio* is morphologically distinct from both other *Euplaesiobystra* species, being different in size, and the only one known to have a cytostome. The amoeboid *E. hypersalinica* and *E. dzianiensis* are 19–41 μm and 17–34 μm in length, and 9–16 μm and 8–21 μm in width (Park et al., 2009; Aucher et al., 2020), respectively, which is larger than our isolate (11.5–23.2 μm long and 5.1–14.5 μm wide). Also, the average diameters of *E. hypersalinica* and *E. dzianiensis* cysts are reported to be 16.8 and 9.5 μm, respectively, which differs from those of our isolate (12.5 μm). Unlike *E. salpumilio*, the flagellate form of *E. hypersalinica* has no clear cytostome (or rostrum), while *E. dzianiensis* has no known flagellate form. The relatively low 18S rDNA identity between *E. salpumilio* and both other species (<85%) also strongly supports *E. salpumilio* being a distinct species. Above all, our isolate is a halophilic heterolobosean species that is physiologically different from marine and freshwater heteroloboseans. Given these similarities and differences, our isolate appears to be a novel species of the genus *Euplaesiobystra*.

The taxonomic assignment of *E. salpumilio* is robustly supported by molecular sequencing data. The presence of the helix 17_1 indicates that *E. salpumilio* appears to be a heterolobosean species (Nikolaev et al., 2004; Park et al., 2009). In addition, molecular phylogeny of the 18S rDNA sequences reveals that *E. salpumilio* branches within the genus *Euplaesiobystra* in Heterolobosea. Notably, *E. salpumilio* is closer to the halophilic *E. hypersalinica* than to the halotolerant *E. dzianiensis*. This result suggests that the halophilic *E. hypersalinica* and *E. salpumilio* have descended from a common halophilic ancestor. Recently, Lee et al. (2022a) notes that *Euplaesiobystra*-like organisms are more diverse in a Eui-Seong solar saltern (the same as our study area) than previously thought. Thus, the taxonomic inventory of *Euplaesiobystra* is still far from complete, and continuous discovery of *Euplaesiobystra* is needed to better understand its evolutionary history. Considering the results from our morphological and molecular phylogenetic data, we propose *E. salpumilio* as a novel species in *Euplaesiobystra*.

### Accumulation of intracellular salts

*E. salpumilio* sp. nov., shows increased levels of intracellular Na^+^ with increasing salinity. The intracellular Na^+^ concentration significantly increased in the cytoplasm of *E. salpumilio* at 100 and 150 PSU compared to that at 50 PSU, in which cells do not grow but are in a viable state. Although Kogej et al. (2005) reports that the Na^+^ ion is a toxic substance for most cells, halophilic *E. salpumilio* clearly accumulates Na^+^ even while actively growing. Thus, it is possible that actively growing *E. salpumilio* under high-salinity conditions requires higher Na^+^ levels than the no-growth but-viable state under low-salinity conditions. This is a new observation for protozoa. Na^+^ requirement is thought to be a specific strategy for salt adaptation, as observed in a halophilic bacterium *Halobacteroides* and a halotolerant yeast *Debaryomyces* (Rengpipat et al., 1988; Prista et al., 2005).

The best-studied halophilic protozoa appear to accumulate compatible solutes (e.g., glycine betaine, ectoine, and hydroxyectoine), and show a salt-out strategy for osmoregulation (Harding et al., 2016; Harding and Simpson, 2018; Weinisch et al., 2018). Yet, they also may show comparatively high intracellular salt concentrations under high-salinity conditions. Harding et al. (2016) suggests that halophilic *Pharyngomonas kirbyi* and *Halocafeteria seosinensis* may have higher intracellular salts than marine relatives, based on the indirect evidence of the hydrophilicity of their proteomes (as inferred through transcriptome analysis). More directly, the halophilic ciliate *Schmidingerothrix salinarum*, which can optimally grow at 90 PSU (growth range: 10–210 PSU), displays an increased intracellular Na^+^ concentration at 210 PSU, as determined using a specific fluorescent dye (i.e., CoroNa Green, Weinisch et al., 2018). This marked increase is inferred to result from the failure to expel the salt. In the present study, the fluorescence intensities representing intracellular Na^+^ at 100 PSU and 150 PSU are not different from each other (Figure 6A), suggesting that *E. salpumilio* actively extrudes the Na^+^ from cells, shifted to 150 PSU, rather than the failure of salt extrusion. Furthermore, *E. salpumilio* can accumulate the intracellular Na^+^ in vacuole-like structures at external salinities of 100 PSU and 150 PSU. Assaha et al. (2017) reports that high retention of sequestered Na^+^ in the vacuole may indicate the enhancement of salt tolerance in plants. Probably, the enhancement of the vacuolar Na^+^ retention is of paramount importance for the survival of *E. salpumilio* in high-salinity waters. Thus, our results suggest that Na^+^-expulsion or retention mechanisms (e.g., Na^+^/H^+^ exchanger and Na^+^/K^+^-ATPase) may be active under high-salinity conditions (Nass and Rao, 1999; Lee et al., 2022b). Actually, it seems that *E. salpumilio* employs two salt metabolisms depending on the tested salinities. Na^+^-retention mechanism of *E. salpumilio* may be active at lower salinity of 100 PSU, whereas Na^+^-expulsion mechanism may be operated at higher salinity of 150 PSU among all tested salinities. Furthermore, this action may allow the tolerance of *E. salpumilio* to higher salinities and maintain growth.

The intracellular K^+^ in the cytoplasm of *E. salpumilio* does not differ significantly between high-salinity media; however, it significantly increases under high-salinity conditions compared to that of the freshwater amoeba *Naegleria neojejuensis*. Furthermore, high retention of intracellular K^+^ is also observed in vacuole-like structures, suggesting that *E. salpumilio* actively takes up K^+^ with increasing salinity. It is possible that some halophilic protozoa simultaneously accumulate intracellular salts, such as Na^+^ and K^+^, regardless of the salt-in or salt-out strategies. Zajc et al. (2014) demonstrated that the intracellular levels of Na^+^ and K^+^ ions increased significantly when halophilic fungus *Wallemia ichthyophaga* was exposed to a hyperosmotic shock from 15% NaCl to 25% NaCl. Thus, we cannot exclude the possibility that *E. salpumilio* may undergo a hyperosmotic shock under hypersaline conditions. Harding and Simpson (2018) speculates that halophilic microbial eukaryotes may have different and diverse strategies for salt adaptation. Therefore, it is reasonable to conclude that *E. salpumilio* fundamentally employs a salt-out strategy at higher salinity of 150 PSU, and that its intracellular ion accumulation patterns are distinct from those of other halophilic microbial eukaryotes. Our study is based on ion imaging measurements, thus further examinations of the transcriptome and quantities of intracellular salts are needed to confirm our findings and provide further insights.

## Data availability statement

The data presented in this study are deposited in the related repositories. The names of the repositories and accession numbers are included in the article. Further inquiries are available on request to the corresponding author.

## Author contributions

JP designed and supervised the study. HL and DJ performed the majority of experiments. HL and JP drafted the manuscript. All authors contributed to the article and approved the submitted version.

## Funding

This work was supported by the National Research Foundation of Korea (NRF) grant, funded by the Korean government to JP (NRF-2022R1I1A2064117), and the National Institute of Biological Resources of Korea (NIBR) grant (NIBR202231206), funded by the Ministry of Environment (MOE) of the Republic of Korea.

## Conflict of interest

The authors declare that the research was conducted in the absence of any commercial or financial relationships that could be construed as a potential conflict of interest.

## Publisher’s note

All claims expressed in this article are solely those of the authors and do not necessarily represent those of their affiliated organizations, or those of the publisher, the editors and the reviewers. Any product that may be evaluated in this article, or claim that may be made by its manufacturer, is not guaranteed or endorsed by the publisher.

## Taxonomy summary

*Assignment*: Eukaryota; Discoba; Discicristata; Heterolobosea; Tetramitia; *Euplaesiobystra.*

*Description*: Trophozoite amoeba with eruptive pseudopodia; 11.5–23.2 μm (average: 17.6 μm) long, 5.1–14.5 μm (average: 9.2 μm) wide; average *L/B* ratio = 2.1; short and fine uroidal filaments; a single nucleus with a single nucleolus.

Flagellate with a spherical shape (9–10 μm diameter); with two flagella of equal length (1.5–2.0 times the body size); the presence of cytostome and rostrum.

Cyst; a spherical shape; 8.3–19.3 μm in diameter (average: 12.5 μm); 2–4 plugged pores; thick endocyst and thin ectocyst; with a smooth outline of ectocyst.

Salinity range for growth from 75 to 150 practical salinity units (PSU) at 35°C.

*Type material*: A DNA sample from a monoprotistan culture of *Euplaesiobystra salpumilio* ES010 was deposited at the National Institute of Biological Resources (NIBR), Incheon, Republic of Korea, as NIBRGR0000645181. This DNA material constitutes the name-bearing hapantotype for the species (see Article 72.5.4 of the International Code of Zoological Nomenclature).

*Type locality*: The type culture ES010 was isolated from hypersaline water with 134 PSU in a Eui-Seong solar saltern, Republic of Korea (36° 36′ 08.3″ N, 126° 17′ 49.3″ E). The temperature of the water was 16.3°C.

*Etymology*: *salpumilio* is a combination of *sal* (= salt in Latin) and *pumilio* (= dwarf in Latin), referring to the small amoeba that inhabits saline environments.

*Gene sequence*: The 18S rRNA gene sequence from the *E. salpumilio* isolate has GenBank accession number ON561780.

*Zoobank registration*: urn:lsid:zoobank.org:act:8E7465F0-1397-4AF7-A92A-06D2CAB12B6E.
